# Supplementary material for: Effects of Sucrose Replacement by Polyols on the Dough-Biscuit Transition: Understanding by Model Systems
Source: Foods. 2023 Feb 1;12(3):607. doi: 10.3390/foods12030607 (PMC9913932; doi:10.3390/foods12030607)
Supplement: Supplementary file 1 [file foods-12-00607-s001.zip › foods-2097933-supplementary.pdf]

Supplementary Material

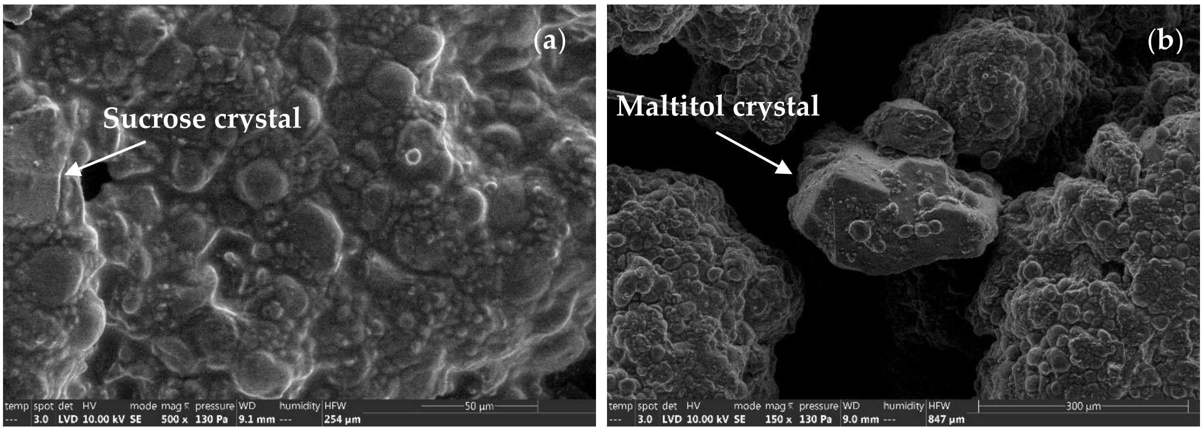

Figure S1. Observation of doughs in ESEM. (**a**): sucrose crystal embedded in dough matrix (white arrow) ×500; (**b**): maltitol crystal among dough particles (white arrow) ×150.
